# Impact of Optical Coherence Tomography Scan Pattern and Chorio-Scleral Interface Delineation on Choroidal Vascularity Index Measurements

**DOI:** 10.1007/s44402-026-00159-4

**Published:** 2026-08-15

**Authors:** Kryshell Yu Qi Wong, Rachel Ka Man Chun, David Alonso-Caneiro, Yan Pui Chan, Andrew Kwok Cheung Lam

**Affiliations:** 1 Centre for Myopia Research, School of Optometry, The Hong Kong Polytechnic University, Kowloon, Hong Kong; 2 Centre for Eye and Vision Research (CEVR), Hong Kong Science Park, Sha Tin, Hong Kong; 3 Research Centre for SHARP Vision (RCSV), The Hong Kong Polytechnic University, Kowloon, Hong Kong; 4 School of Science, Technology and Engineering, University of Sunshine Coast, Petrie, Queensland Australia; 5 Contact Lens and Visual Optics Laboratory, Centre for Vision and Eye Research, Optometry and Vision Science, Queensland University of Technology (QUT), Kelvin Grove, Queensland Australia

**Keywords:** Chorio-scleral interface, Choroid, Choroidal thickness, Choroidal vascularity index, Optical coherence tomography, Scan pattern

## Abstract

**Purpose:**

To compare radial and horizontal raster acquisition patterns using spectral-domain optical coherence tomography (OCT) for assessing choroidal vascularity index (CVI) in healthy eyes and to evaluate how variability in chorio-scleral interface (CSI) delineation influences CVI measurements.

**Methods:**

OCT images of 25 eyes from 25 healthy adults were acquired using 12-line radial and 31-line horizontal raster scans with enhanced depth imaging. CVI was measured manually using three different posterior boundaries—defined as the upper (vascular), middle (vascular–stromal) and lower (stromal) CSI boundaries. Agreement between CSI boundaries and scan patterns was assessed using Bland-Altman analysis. Topographical CVI variations across 6-mm Early Treatment of Diabetic Retinopathy Study (ETDRS) subfields, quadrants and rings were evaluated using two-way repeated-measures ANOVA.

**Results:**

Participants were aged 21–31 years (72% female; mean spherical equivalent −2.97 ± 2.02 D). Mean CVI differences ranged from 1.43 to 2.38% between the upper and middle CSI boundaries and from 1.23 to 2.04% between the middle and lower CSI boundaries. Comparisons between the upper and lower CSI boundaries resulted in larger CVI differences, ranging from 2.90 to 4.42%. The coefficient of variation for CVI was low (<2%) between radial and raster scan patterns, and Bland-Altman analysis showed overall good agreement. The 31-line raster scan measured 0.51% higher CVI in the central foveal region, whereas the 12-line radial scan yielded 0.64–1.10% higher CVI in the perifoveal region. Significant mean differences were observed only in the outer temporal subfield (−1.10%, *p* = 0.005) and outer ring (−0.77%, *p* = 0.02). No significant scan-pattern effect was found, but CVI varied significantly across the ETDRS grid, with the lowest values in the outer nasal subfield, nasal quadrant and outer ring.

**Conclusions:**

Radial scans provide CVI measurements comparable to raster scans, with faster acquisition, supporting practical use in children. Standardising CSI delineation is crucial for consistent CVI comparisons across studies.

Key Points

- Establishing a standardised chorio-scleral interface definition is essential to ensure consistent, reproducible measurements and to facilitate reliable comparisons across choroidal vascularity index studies.
- Choroidal vascularity index measurements obtained from the 12-line radial scan were comparable to those from the 31-line horizontal raster scan, while requiring only half the acquisition time. This suggests that the radial scan may be a more practical option for routine clinical use, particularly in paediatric patients.
- Compared to choroidal thickness, the choroidal vascularity index showed lower variability between the two scan patterns across the Early Treatment of Diabetic Retinopathy Study regions. This indicates that the choroidal vascularity index is less influenced by scan pattern and may serve as a more stable parameter for choroidal assessment in future research.

## Introduction

The choroid is a densely vascularised layer located between the retina and the sclera. With the advent of enhanced depth imaging optical coherence tomography (EDI-OCT) and OCT angiography, non-invasive assessment of the choroid has become feasible with improved visualisation and accuracy [1,2]. Various choroidal parameters, including choroidal thickness (ChT), choroidal vascularity index (CVI), blood flow and perfusion, have been studied extensively in both healthy and pathological eyes [3–6]. Among these, CVI has emerged as a novel quantitative metric for assessing choroidal vascular status by quantifying the relative proportions of vascular luminal and interstitial stromal components. It was initially termed the luminal-to-choroidal area ratio (L/C ratio) by Sonoda et al. [7], who first proposed the image binarisation technique to differentiate luminal and stromal areas. CVI, later introduced by Agrawal et al. [4], was defined as the ratio of the luminal area (LA) to the total choroidal area (TCA). Growing evidence suggests that CVI may serve as a potential biomarker in a variety of ocular diseases, including age-related macular degeneration [8], diabetic retinopathy [9], polypoidal choroidal vasculopathy [10], panuveitis [11], central serous chorioretinopathy [12], Vogt-Koyanagi-Harada disease [13] and inherited retinal dystrophies [14,15]. However, reported CVI values in healthy populations vary widely, ranging from approximately 45–70% within a 1.0–1.5 mm wide subfoveal choroidal region centred at the fovea [16–19]. There has also been controversy regarding the relationship between CVI, myopia and axial length (AL). While some studies have reported higher CVI values with increasing myopia or longer AL [20–22], others have observed lower CVI values [23–25] or no significant association [26,27].

These conflicting findings may be partly attributable to the lack of a standardised CVI measurement protocol. Variations in OCT devices, scan patterns and definitions of the chorio-scleral interface (CSI) across studies may contribute to such discrepancies. Although Agrawal et al. [28] reported good agreement between spectral-domain OCT (SD-OCT) and swept-source OCT (SS-OCT) for CVI measurements, the potential impact of scan pattern and CSI delineation on CVI values remains largely unexplored. The CSI is commonly used to describe the posterior boundary of the choroid, yet its precise definition remains inconsistent in the literature. While not explicitly stated, Sonoda et al. [7] appear to have adopted the outer border of the choroidal vessels as the CSI for L/C ratio measurements, whereas Agrawal et al. [4] seem to have used the outer border of the choroidal stroma as the posterior boundary of the choroid for CVI measurements, based on visual inspection of their published binarised OCT images. In some eyes, a hyporeflective band observed between the choroidal stroma and sclera, likely corresponding to the suprachoroidal layer (SCL), has also been considered the CSI [29]. Previous research has shown that ChT measured using these different posterior boundaries can vary significantly by up to 70 μm [29]. Such variability is also expected to influence CVI measurements. Therefore, this study aimed to explore how different CSI boundary definitions affect CVI values.

In addition, most existing CVI studies have relied on horizontal raster scan patterns for OCT image acquisition [4,17,19,22,25], whereas relatively few have employed radial scan patterns [23,30,31]. Due to their geometric configuration, raster scans potentially cover a larger area than radial scans as their four corners extend beyond the peripheral boundaries of radial scans [32,33]. Nevertheless, previous studies have indicated that 6-line radial [32] and 12-line radial scans [33] are statistically comparable to 25-line horizontal raster scans in detecting macular diseases, while requiring only one-third or one-half of the acquisition time. Hence, this study also aimed to compare CVI values obtained from radial and horizontal raster scan patterns using SD-OCT and to evaluate the topographical distribution of CVI in healthy eyes based on the Early Treatment of Diabetic Retinopathy Study (ETDRS) grid.

## Methods

### Study Design and Population

This prospective, cross-sectional study was conducted at the Centre for Myopia Research, The Hong Kong Polytechnic University. The inclusion criteria were healthy Chinese young adults: (1) aged between 18 and 35 years; (2) spherical equivalent refraction (SER) ranging from −6.00 to +1.00 D, with myopic astigmatism ≤2.00 D and anisometropia ≤1.50 D and (3) best-corrected visual acuity (BCVA) of 0.00 logMAR or better in each eye. Exclusion criteria were individuals with: (1) a history of ocular surgery or trauma; (2) known ocular disorders such as cataract, glaucoma, diabetic retinopathy, uveitis or any chorioretinal disease; (3) systemic diseases such as hypertension or diabetes; (4) strabismus or amblyopia; (5) use of systemic medication that might affect ocular blood flow; (6) intraocular pressure (IOP) > 21 mmHg in either eye; (7) systolic blood pressure (SBP) ≥ 140 mmHg or diastolic blood pressure (DBP) ≥ 90 mmHg; (8) a smoking habit [34] and (9) currently undergoing any myopia control treatment. To minimise potential confounding effects, participants were instructed to refrain from vigorous physical activity [35] and from consuming caffeinated [36,37] and alcoholic [38] drinks for at least 12 h before the study visit. Written informed consent was obtained from each participant prior to their enrolment. This study conformed to the tenets of the Declaration of Helsinki and was approved by the Institutional Review Board of The Hong Kong Polytechnic University (HSEARS20230914006).

### Procedures and Ophthalmic Examination

Demographic information, medical and ocular history were collected from each participant. A series of ophthalmic screening tests was then conducted to confirm their eligibility. Refractive error was measured using an open-field autorefractor (Shin-Nippon NVision-K5001, Rexxam, rexxam.co.jp), followed by non-cycloplegic subjective refraction. BCVA was assessed monocularly using the logMAR letters on the Thomson Test Chart XPert 3Di (Thomson Software Solutions, thomson-software-solutions.com) at 6 metres. Five autorefraction readings with a maximum-minimum deviation of ≤0.25 D were obtained for each eye. IOP was measured using a non-contact tonometer (CT-80 Computerised Tonometer, Topcon Healthcare, Inc., topconhealthcare.com). Anterior segment health was evaluated by slit lamp biomicroscopy (SL-D701, Topcon Healthcare, Inc., topconhealthcare.com), and posterior segment health was assessed via fundus photography (DRI OCT Triton, Topcon Healthcare, Inc., topconhealthcare.com). Blood pressure, including SBP and DBP, was measured using a digital blood pressure monitor (HEM-7156, Omron Healthcare, omronhealthcare-ap.com). Eligible participants were instructed to rest and watch a movie displayed at a distance of 6 metres for 15 min while wearing their full-distance refractive correction, in order to minimise the potential influence of physical activity and prior visual stimuli such as optical defocus [39,40] or accommodation [41,42] on the choroid. Prior to OCT acquisition, AL was measured using a non-contact partial coherence interferometer (ZEISS IOL Master 500, Carl Zeiss Meditec AG, zeiss.com). Five AL readings were taken, with a maximum-minimum deviation of ≤0.02 mm.

### OCT Measurements

All SD-OCT scans were performed using the Heidelberg Spectralis OCT2 (Heidelberg Engineering, heidelbergengineering.com). This device utilises a superluminescent diode with a central peak wavelength of around 870 nm and operates at a scan rate of 85,000 A-scans per second, providing axial and transverse tissue resolutions of 3.9 and 14 μm, respectively. Choroidal imaging was performed by an experienced examiner (KW) using two scanning protocols: a 12-line radial scan covering an area of 30° (9 mm), followed by a 31-line horizontal raster scan covering 30° × 25° (9 mm × 7.5 mm), both centred on the fovea. The imaging protocol employed enhanced depth imaging (EDI) and high-speed modes, with eye-tracking Automatic Real-Time averaging set to 35. The acquisition time was approximately 6–8 s for the 12-line radial scan and 12–15 s for the 31-line raster scan. Prior to image acquisition, keratometry and refraction data of each participant were entered into the Spectralis built-in HEYEX (Heidelberg Eye Explorer) software to correct for ocular magnification [43]. The eye size setting (medium, large or extra large) was also modified at the acquisition window until achieving a clear focus on the retina. Participants were instructed to fixate on a central blue light target during the OCT examination. All scans were performed on the right eye under dim illumination without pupil dilation. Scans were repeated by the same examiner (KW) if they did not meet any of the following criteria: (1) image signal strength ≥20 dB; (2) clear visualisation of large choroidal vessels in Haller’s layer and a well-defined CSI, free of artefacts such as media opacities or shadowing from overlying retinal vasculature and (3) no significant B-scan tilting. To be considered successful, all B-scans in both the 12-line radial and 31-line raster scans had to meet these criteria. All OCT measurements were conducted between 09:00 and 13:00 h to minimise the influence of diurnal variation on choroidal parameters [44,45].

### CVI Measurements

CVI was measured manually by two experienced examiners (KW and PC) using the publicly available ImageJ software, version 1.53t (National Institutes of Health, imagej.nih.gov/ij). After loading the selected OCT B-scan image into ImageJ, the scale setting was performed by converting pixel measurements to micrometres (μm) using the transverse scale (Size X or Scaling X) provided by the Spectralis system for each OCT scan of each participant. The pixel aspect ratio was also adjusted to account for the ratio difference between the vertical and horizontal scales in the 1:1 pixel image [46]. Next, the choroidal region of interest (ROI) was first delineated on the grayscale OCT image using the polygon tool and added to the ROI manager. The OCT scan was then converted to an 8-bit image utilising the default setting in ImageJ, and the Niblack auto local threshold tool was applied to binarise the image. After image binarisation, the CSI boundary of the initially selected ROI was adjusted where necessary and added to the ROI manager, thereby highlighting the TCA. The image was then converted to red-green-blue (RGB) format, and the colour threshold tool was used to distinguish the dark pixels, representing the LA. The stromal area (SA), referred to as the area of light pixels, was calculated by subtracting LA from TCA. CVI was calculated as the ratio of LA to TCA and expressed as a percentage.

#### CSI Delineation

**Fig. 1:**
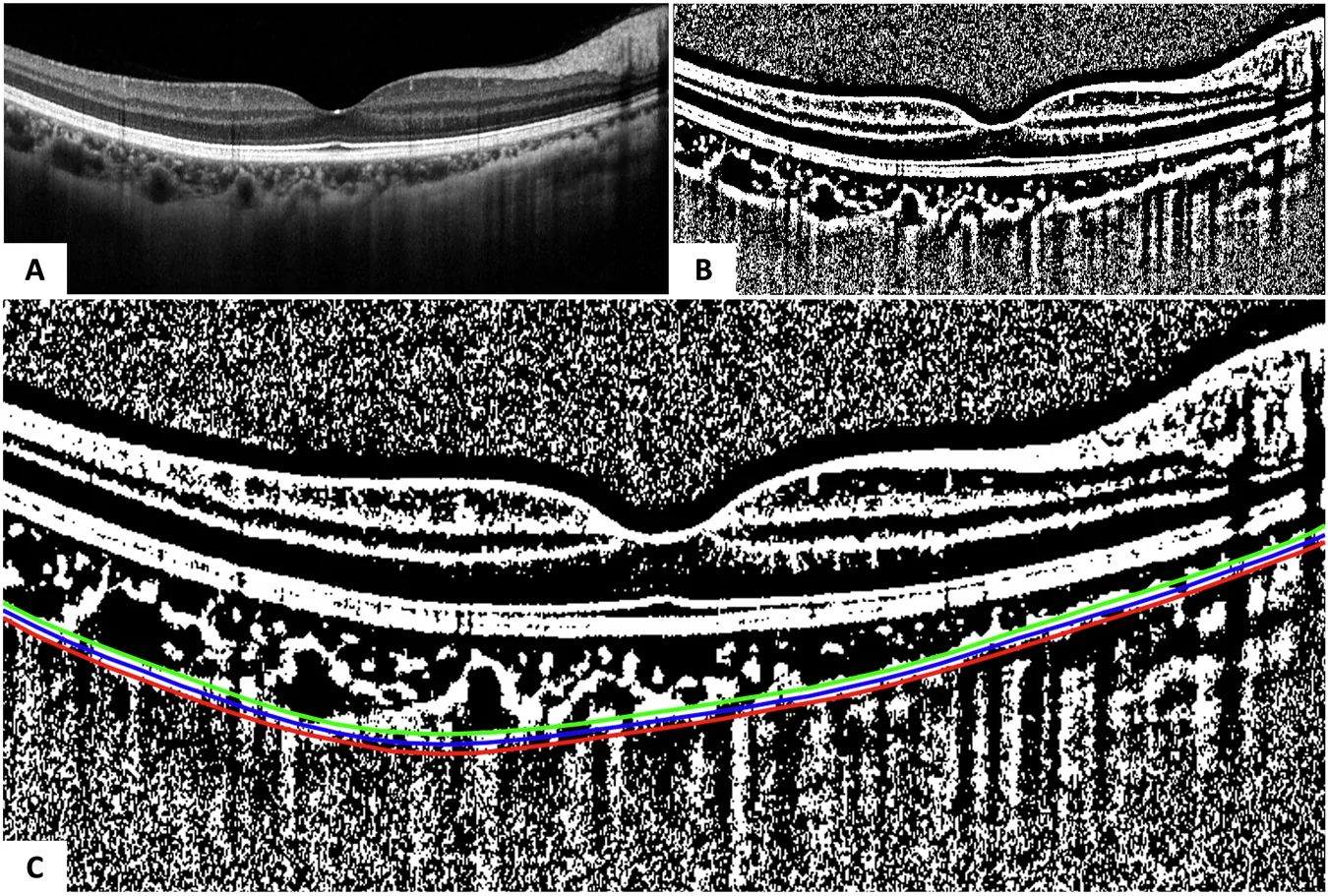
Chorio-scleral interface (CSI) delineation. Example of **A** an original optical coherence tomography (OCT) B-scan and **B** corresponding binarised image. **C** Delineation of the chorio-scleral interface (CSI) using three different posterior boundaries: the upper (green line), middle (blue line) and lower (red line) CSI boundaries.

To examine how variability in CSI definition influences CVI values, Examiner 1 (KW) performed CVI measurements on all OCT B-scans from the 12-line radial scans using three distinct boundaries: the upper, middle and lower CSI boundaries (Fig. 1). The ‘upper CSI boundary’ corresponds to the outer border of the hyporeflective choroidal vessels (vascular choroid), as adopted by Sonoda et al. [7]; the ‘lower CSI boundary’ corresponds to the outer border of the hyperreflective choroidal stroma (stromal choroid), as adopted by Agrawal et al. [4] and the ‘middle CSI boundary’ corresponds to the vascular–stromal choroid, located midway between the upper and lower CSI boundaries. Examiner 2 (PC), who was masked to the participants’ demographic and clinical details, independently performed CVI measurements on the same set of OCT images using only the middle CSI boundary. Although this boundary may appear arbitrary and involve examiner bias due to subjective judgement of its exact location, between-examiner agreement was excellent, with intraclass correlation coefficients ranging from 0.943 to 0.987. Therefore, CVI measurements obtained using the middle CSI boundary from both examiners were averaged and used for subsequent analyses comparing radial and raster scan patterns.

#### ETDRS-Based CVI Map

To facilitate comparisons between radial and raster scan patterns, CVI was measured based on a 6-mm ETDRS grid, in which the macular region is divided into nine subfields (C, S1, I1, T1, N1, S2, I2, T2 and N2), defined by three rings (1 mm central (C) foveal ring, 1–3 mm inner ring and 3–6 mm outer ring) and four quadrants (superior (S), inferior (I), temporal (T) and nasal (N)). An ETDRS-based CVI map was generated by averaging CVI values from the scan lines corresponding to each ETDRS subfield for both scan patterns.

**Fig. 2:**
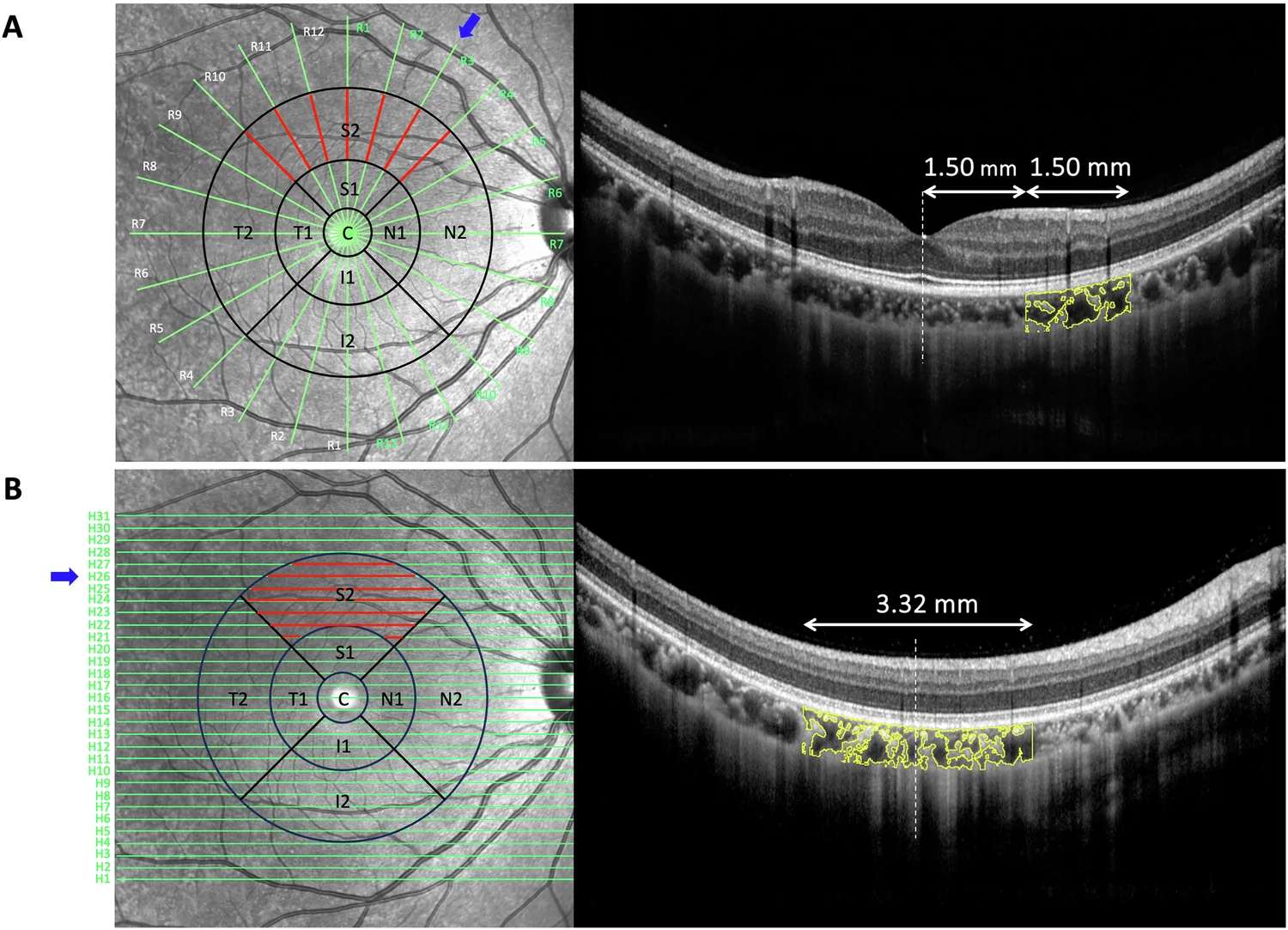
Extraction of choroidal vascularity index (CVI) based on a 6-mm Early Treatment of Diabetic Retinopathy Study (ETDRS) grid. Extraction of choroidal vascularity index (CVI) based on a 6-mm Early Treatment of Diabetic Retinopathy Study (ETDRS) grid. For instance, the mean CVI of the S2 subfield was calculated by averaging the CVI values from the seven red scan lines in both the **A** (left) 12-line radial and **B** (left) 31-line horizontal raster scans. Examples of OCT B-scan image from the **A** (right) 12-line radial scan (R3 scan line, indicated by blue arrow) and the **B** (right) 31-line horizontal raster scan (H26 scan line, indicated by blue arrow), with their corresponding regions of interest (yellow overlay) used for CVI measurements in the S2 subfield. The white dashed line indicates the centre of the fovea. C central, I inferior, N nasal, S superior, T temporal.

For the 12-line radial scan (Fig. 2A), the CVI of the central subfield was calculated as the average CVI of all twelve radial lines [R1–R12], where the ROI on each B-scan image was 1.0 mm centred over the fovea. In the other eight subfields, each subfield was contributed by the average CVI from seven radial lines passing through the specific subfield, with ROI varying across different subfields. For example, the CVI of the S2 subfield was the average CVI of the superior halves of R1–R4 and R10–R12 scan lines, with the ROI being 1.50 mm, located 1.50 mm away from the fovea centre. Similar concepts were applied to the other subfields for the radial scan.

For the 31-line raster scan (Fig. 2B), which is not inherently compatible with the circular design of the ETDRS grid, a series of mathematical calculations were performed to determine the number of horizontal lines and their respective ROIs contributing to the CVI of each subfield. In the central subfield, the mean CVI was the average CVI of the three middle horizontal raster lines [H15, H16 and H17], with the ROIs being 0.866, 1.000 and 0.866 mm, respectively, centred over the fovea. Similarly, the CVI of the S2 subfield was derived from the average CVI of H21–H27, with ROIs ranging from 0.842 to 4.000 mm. Table 1 presents the total number of data points (i.e., pixels, calculated from the cumulative lengths of the ROIs) and the total number of B-scans used for CVI measurements in each ETDRS subfield across the two scan patterns.

**Table 1:** Total number of pixels and B-scans (in square brackets) used for choroidal vascularity index (CVI) measurements in each Early Treatment of Diabetic Retinopathy Study (ETDRS) subfield across the two scan patterns.

ETDRS	Total number of pixels [Total number of B-scans]	
	12-line radial	31-line raster
C	1024 [12]	233 [3]
S1	597 [7]	526 [4]
I1	597 [7]	526 [4]
T1	597 [7]	534 [9]
N1	597 [7]	534 [9]
S2	896 [7]	1830 [7]
I2	896 [7]	1830 [7]
T2	896 [7]	1812 [17]
N2	896 [7]	1812 [17]

Scale: 768 pixels (9.0 mm); 1 pixel: 0.01171875 mm.

*C* central, *I* inferior, *N* nasal, *S* superior, *T* temporal.

Other than subfield CVI, the CVI of each ETDRS quadrant and ring was also explored in this study. CVI in the superior quadrant was calculated as the average CVI of the S1 and S2 subfields, whereas in the inner ring, it was the average CVI of the S1, I1, T1 and N1 subfields. Similar calculations were applied to other quadrants and rings.

### ChT Measurements

ChT was defined as the perpendicular distance between the outer border of the hyperreflective retinal pigment epithelium-Bruch’s membrane complex and the CSI. All OCT B-scan images from the 12-line radial and 31-line horizontal raster scans were exported and processed using a previously validated custom deep-learning software for automated segmentation and ChT measurement [47,48]. While not clearly mentioned in prior studies, visual inspection of the published segmented images [48] suggests that the software defines the CSI using the outer border of the choroidal vessels—referred to in this study as the ‘upper CSI boundary’. To ensure consistency when comparing the effects of scan patterns on both ChT and CVI, each automatically segmented B-scan was carefully reviewed by an experienced examiner (KW) and the CSI boundary was manually adjusted from the upper to the middle CSI boundary. The software then automatically generated ChT values for each subfield of the 6-mm ETDRS grid, which were exported to a Microsoft Excel (Microsoft.com) file for further analysis.

### Statistical Analysis

Based on previous studies, the average CVI in healthy individuals of a similar age range is approximately 62% when measured using a raster scan [7,21,22,49,50] and around 58% when using a radial scan [23,30,31,51,52]. This study aimed to detect at least a 1% difference between the two scan patterns, assuming a standard deviation (SD) of differences of 1.75%. A power analysis using G*Power software (version 3.1.9.6; psychologie.hhu.de) indicated that a minimum sample size of 24 participants would be required to achieve 80% statistical power at a two-tailed significance level of 0.05.

Only data from the right eyes of each participant were analysed. The Shapiro-Wilk test was used to assess the normality of the data. Agreement among CVI values derived from the upper, middle and lower CSI boundaries was examined using Bland-Altman analysis, and paired samples *t*-tests with Bonferroni correction were performed for statistical comparisons. Agreement between scan patterns for CVI and ChT measurements was also evaluated using Bland-Altman analysis. The coefficient of variation (CoV) was calculated to determine the relative variability of CVI and ChT between scan patterns. Three separate two-way repeated-measures analysis of variance (RM-ANOVA) models were conducted to compare the topographical variations of CVI across the ETDRS grid between the two scan patterns. In Model 1, the within-subject factors were scan pattern (two levels: 12-line radial, 31-line raster) and subfield (nine levels: C, S1, I1, T1, N1, S2, I2, T2, N2). In Model 2, the factors were scan pattern and quadrant (four levels: superior, inferior, temporal, nasal). In Model 3, the factors were scan pattern and ring (three levels: central foveal, inner and outer). Post-hoc analysis with Bonferroni correction was performed where significant main or interaction effects were found. All statistical analyses were performed using IBM SPSS software (version 29.0; ibm.com).

## Results

A total of 25 eyes from 25 participants were included in the analysis. Demographic and baseline characteristics are summarised in Table 2. The mean age of participants was 26.72 ± 2.75 years, with 72% being female, and the mean SER was −2.97 ± 2.02 D.

**Table 2:** Demographic and baseline characteristics of study participants (*n* = 25).

Baseline data	Mean ± SD	Range
Age, years	26.72 ± 2.75	21–31
Sex, *n* (%)		
Male	7 (28.0%)	
Female	18 (72.0%)	
SER, D	−2.97 ± 2.02	−6.00 to +1.00
Axial length, mm	24.58 ± 1.06	22.57–26.34
CR, mm	7.75 ± 0.23	7.30–8.24
IOP, mmHg	15.40 ± 2.04	11.50–19.00
Systolic BP, mmHg	108.64 ± 12.14	90–128
Diastolic BP, mmHg	77.48 ± 8.21	62–90

*BP* blood pressure, *CR* corneal radius of curvature, *D* dioptre, *IOP* intraocular pressure, *SD* standard deviation, *SER* spherical equivalent refraction.

### Upper vs. Middle vs. Lower CSI Boundary

**Fig. 3:**
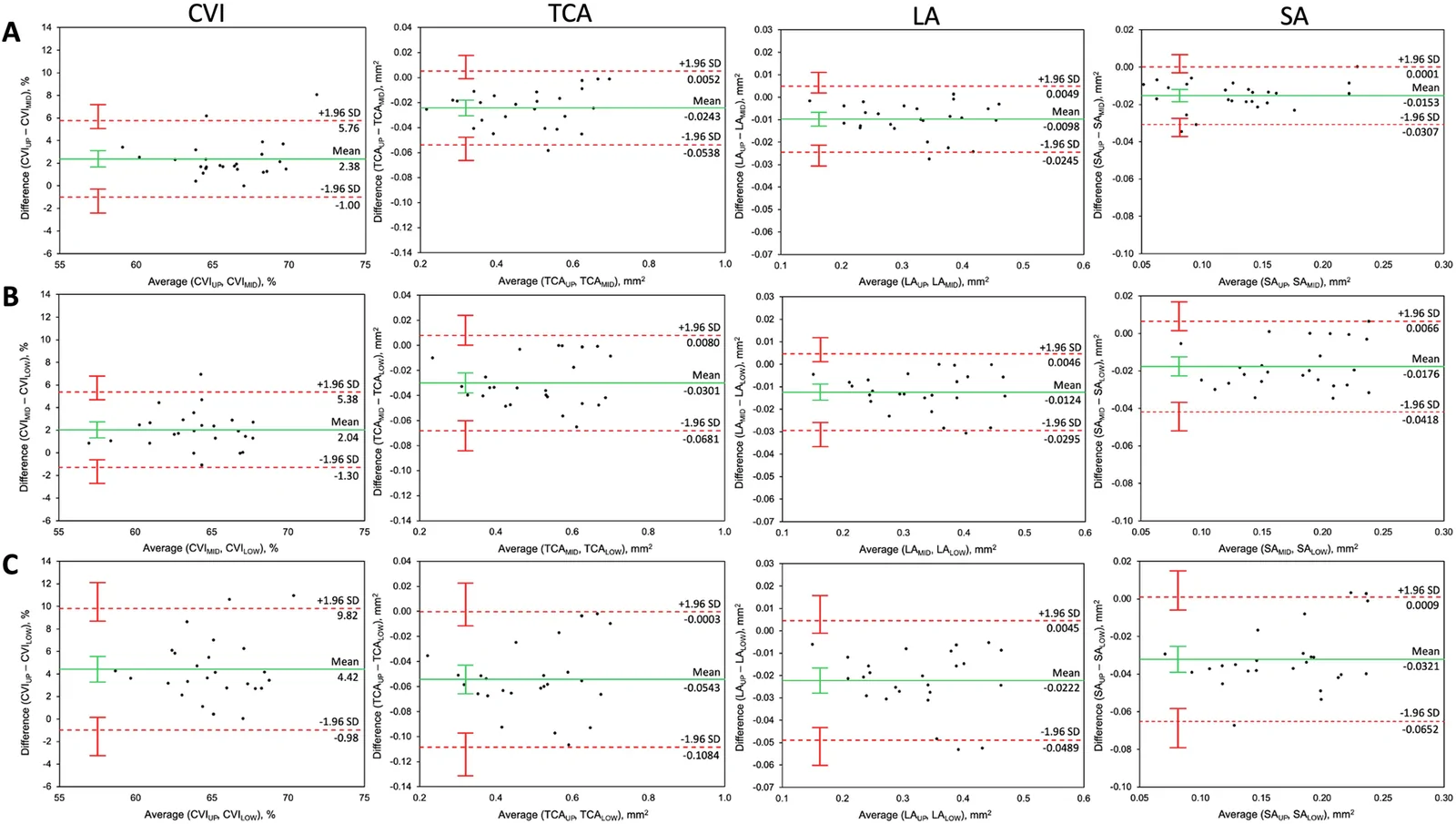
Agreement between different chorio-scleral interface (CSI) boundaries. Bland-Altman plots showing agreement between **A** upper (UP) vs. middle (MID) CSI, **B** middle vs. lower (LOW) CSI and **C** upper vs. lower CSI boundaries for CVI, TCA, LA and SA measurements in the outer nasal subfield. The green solid line indicates the mean difference, and the red dashed lines represent the upper and lower limits of agreement. Each error bar denotes the 95% confidence interval for the mean difference and the limits of agreement. CVI choroidal vascularity index, LA luminal area, SA stromal area, TCA total choroidal area.

The mean CVI values obtained using the upper, middle and lower CSI boundaries were 67.73 ± 3.11, 65.89 ± 2.43 and 64.26 ± 2.22%, respectively. Bland-Altman analysis across the 6-mm ETDRS regions showed mean differences between the upper and middle CSI boundaries for CVI (range: 1.43–2.38%), TCA (range: −0.0243 to −0.0125 mm^2^), LA (range: −0.0098 to −0.0045 mm^2^) and SA (range: −0.0153 to −0.0080 mm^2^) (Fig. 3A). Similar ranges of mean differences were observed between the middle and lower CSI boundaries: CVI (1.23–2.04%), TCA (−0.0301 to −0.0138 mm^2^), LA (−0.0124 to −0.0060 mm^2^) and SA (−0.0176 to −0.0074 mm^2^) (Fig. 3B). Comparisons between the upper and lower CSI boundaries revealed even greater mean differences in CVI (2.90 to 4.42%), TCA (−0.0543 to −0.0273 mm^2^), LA (−0.0222 to −0.0105 mm^2^) and SA (−0.0321 to −0.0163 mm^2^) (Fig. 3C). The largest discrepancies were consistently noted in the N2 subfield. All pairwise differences in CVI (Table 3), TCA, LA and SA (Supplementary eTables 1–3) across the three CSI boundaries were statistically significant (all *p* < 0.01). These results suggest that CVI measurements are significantly influenced by how the CSI boundary is defined.

**Table 3:** Mean differences in CVI derived from three different CSI boundaries.

ETDRS	Mean difference [95% LoA]		
	Upper vs. middle CSI	Middle vs. lower CSI	Upper vs. lower CSI
C	2.12 [−1.88 to 6.13]	1.34 [−1.95 to 4.62]	3.46 [−3.12 to 10.04]
S1	1.66 [−1.72 to 5.04]	1.23 [−2.21 to 4.67]	2.90 [−3.13 to 8.93]
I1	1.70 [−1.83 to 5.24]	1.59 [−2.35 to 5.53]	3.30 [−3.30 to 9.89]
T1	1.85 [−1.82 to 5.43]	1.75 [−1.24 to 4.74]	3.56 [−2.36 to 9.48]
N1	2.03 [−1.50 to 5.57]	1.99 [−1.52 to 5.51]	4.03 [−2.20 to 10.25]
S2	1.68 [−0.62 to 3.99]	1.23 [−1.31 to 3.76]	2.91 [−0.71 to 6.53]
I2	1.43 [−0.94 to 3.80]	1.59 [−1.17 to 4.36]	3.03 [−1.45 to 7.50]
T2	1.73 [−1.71 to 5.18]	1.84 [−1.39 to 5.07]	3.57 [−1.77 to 8.92]
N2	2.38 [−1.00 to 5.76]	2.04 [−1.30 to 5.38]	4.42 [−0.98 to 9.82]

*C* central, *CSI* chorio-scleral interface, *CVI* choroidal vascularity index, *ETDRS* Early Treatment of Diabetic Retinopathy Study, *I* inferior, *LoA* limits of agreement, *N* nasal, *S* superior, *T* temporal.

All pairwise differences were statistically significant, paired samples *t*-tests with Bonferroni correction, *p* < 0.01.

### Radial vs. Raster OCT Scan Pattern

Table 4 presents the mean CVI values obtained from the 12-line radial and 31-line raster scan patterns across the ETDRS subfields, quadrants and rings. The overall CoV was low (all <2%), indicating minimal variation between the two scan patterns. Bland-Altman analysis demonstrated generally good agreement in CVI measurements between the 12-line radial and 31-line raster scans across the ETDRS grid, with larger mean differences noted in the central and outer subfields and rings. Specifically, the 31-line raster scan tended to measure 0.51% higher CVI values than the 12-line radial scan at the central subfield and ring, while the 12-line radial scan tended to produce higher CVI values (range: 0.64–1.10%) in the outer subfields (S2, I2, T2, N2) and the outer ring. Despite these trends, statistically significant mean differences were found only in the T2 subfield (mean bias: −1.10%, 95% LoA: −4.58 to 2.39%, *p* = 0.005) and the outer ring (mean bias: −0.77%, 95% LoA: −3.64 to 2.10%, *p* = 0.02).

**Table 4:** Mean choroidal vascularity index values and the degree of variation and agreement between the 12-line radial and 31-line raster scan patterns across the Early Treatment of Diabetic Retinopathy Study (EDTRS) subfields, quadrants and rings.

ETDRS	Choroidal vascularity index, %			
	Mean ± SD		CoV, %	Bland-Altman
	12-line radial	31-line raster		Mean difference [95% LoA]
*Subfields*				
C	65.46 ± 2.26	65.98 ± 2.11	1.81	0.51 [−3.82 to 4.84]
S1	65.67 ± 2.07	65.55 ± 1.54	1.83	−0.12 [−4.43 to 4.19]
I1	66.66 ± 2.19	66.57 ± 2.70	1.76	−0.09 [−4.36 to 4.18]
T1	66.34 ± 2.31	66.25 ± 2.25	1.69	−0.09 [−3.79 to 3.62]
N1	66.50 ± 2.68	66.50 ± 2.34	1.77	−0.01 [−4.04 to 4.02]
S2	65.06 ± 2.05	64.37 ± 1.42	1.55	−0.69 [−4.02 to 2.64]
I2	65.62 ± 2.03	64.97 ± 1.34	1.71	−0.65 [−4.40 to 3.10]
T2	66.21 ± 2.04	65.11 ± 1.57	1.75	−1.10 [−4.58 to 2.39]**
N2	64.73 ± 2.93	64.09 ± 2.66	1.70	−0.64 [−4.43 to 3.15]
*Quadrants*				
Superior	65.62 ± 2.57	65.29 ± 2.12	1.48	−0.32 [−3.54 to 2.90]
Inferior	65.90 ± 2.14	65.51 ± 1.51	1.61	−0.38 [−3.58 to 2.82]
Temporal	66.27 ± 1.93	65.68 ± 1.68	1.42	−0.59 [−3.71 to 2.53]
Nasal	65.36 ± 1.85	64.96 ± 1.29	1.48	−0.41 [−3.84 to 3.03]
*Rings*				
Central foveal	65.46 ± 2.26	65.98 ± 2.11	1.81	0.51 [−3.82 to 4.84]
Inner	66.29 ± 2.23	66.22 ± 1.60	1.54	−0.07 [−3.25 to 3.39]
Outer	65.40 ± 1.89	64.63 ± 1.29	1.39	−0.77 [−3.64 to 2.10]*

*C* central, *CoV* coefficient of variation, *I* inferior, *LoA* limits of agreement, *N* nasal, *S* superior, *SD* standard deviation, *T* temporal.

Statistical significance was determined using a paired samples *t*-test, *p < 0.05, **p < 0.01.

The mean ChT values obtained from the 12-line radial and 31-line raster scan patterns are presented in Supplementary eTable 4. The CoV ranged from 2.84 to 19.10%, indicating moderate to large variation in ChT between the two scan patterns. Bland-Altman analysis revealed poor agreement in ChT measurements between the 12-line radial and 31-line raster scans, with significant mean differences observed across all ETDRS regions, ranging from 4.10 to 61.91 μm. These findings suggest that CVI may be less affected by the OCT scan pattern than ChT.

**Fig. 4:**
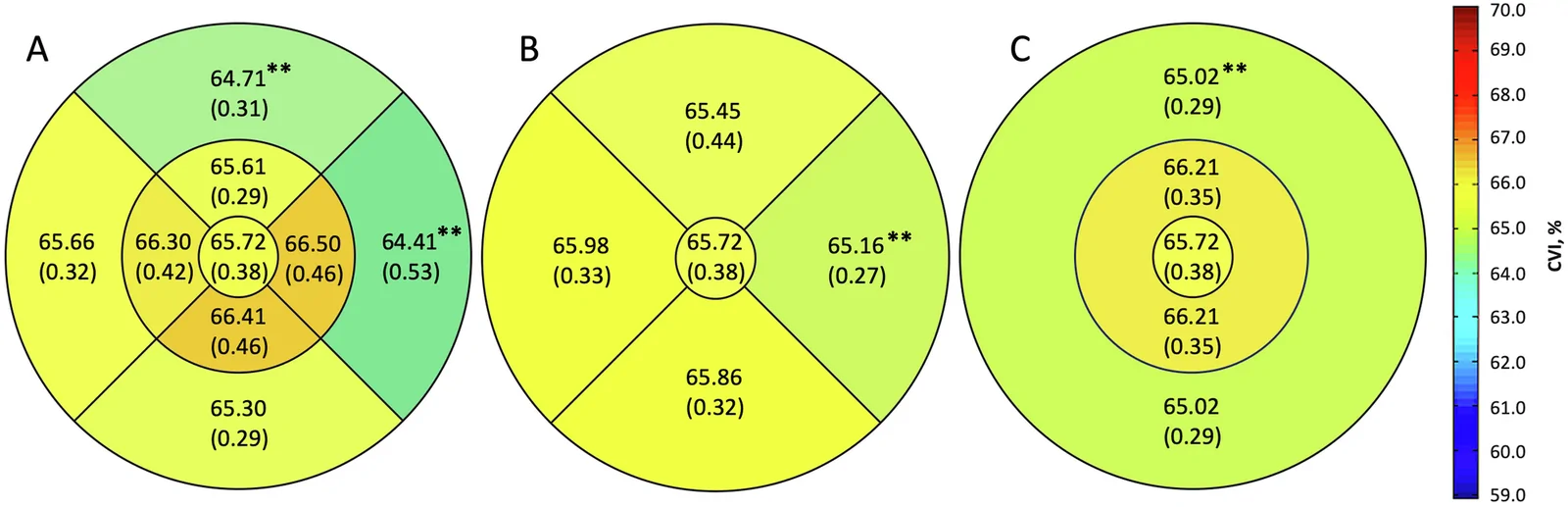
Topographical distribution of choroidal vascularity index (CVI). Colour-coded maps illustrating the estimated marginal means (standard error) of choroidal vascularity index (CVI) across the Early Treatment of Diabetic Retinopathy Study (ETDRS) **A** subfields, **B** quadrants and **C** rings. Statistically significant differences in CVI between subfields, quadrants or rings were identified using two-way repeated-measures ANOVA with post-hoc Bonferroni correction, **p < 0.01. ANOVA analysis of variance.

A two-way repeated-measures ANOVA revealed no significant main effect of scan pattern on regional variations in CVI across all three models (Table 5). CVI exhibited significant topographical variation across ETDRS subfields (Model 1: *F* = 7.148, *p* < 0.001), quadrants (Model 2: *F* = 3.306, *p* = 0.04) and rings (Model 3: *F* = 8.838, *p* < 0.001). The estimated marginal means of CVI in various subfields, quadrants and rings are illustrated in Fig. 4 and Supplementary eTable 5. Post-hoc comparisons indicated that the S2 and N2 subfields had significantly lower CVI than all other subfields (all *p* < 0.05). No significant difference was observed between the S2 and N2 subfields. The nasal quadrant exhibited significantly lower CVI compared to both the inferior (*p* = 0.003) and temporal quadrants (*p* = 0.004). Additionally, CVI in the outer ring was significantly smaller than in the central foveal (*p* = 0.045) and inner rings (*p* < 0.001).

**Table 5:** Within-subject effects from two-way repeated-measures ANOVA.

Model	Effects	Sum of squares	df	Mean square	*F*	*p* value	Partial eta squared
1	Scan	7.285	1.000	7.285	0.713	0.41	0.029
	Subfields	213.392	4.402	48.474	7.148	<0.001**	0.229
	Scan × Subfields	30.605	8.000	3.826	4.341	<0.001**	0.153
2	Scan	6.127	1.000	6.127	1.430	0.24	0.056
	Quadrants	21.019	2.104	9.990	3.306	0.04*	0.121
	Scan × Quadrants	1.672	3.000	0.557	1.143	0.34	0.045
3	Scan	0.150	1.000	0.150	0.038	0.8	0.002
	Rings	35.537	2.000	17.769	8.838	<0.001**	0.269
	Scan × Rings	10.565	2.000	5.282	10.973	<0.001**	0.3145

*ANOVA* analysis of variance, *dF* degree of freedom, *F* *F*-ratio from ANOVA.

Bold values indicate statistically significant effects, *p < 0.05, **p < 0.01.

Significant interaction effects were also observed between scan × subfields (Model 1: *F* = 4.341, *p* < 0.001) and between scan × rings (Model 3: *F* = 10.973, *p* < 0.001), as shown in Table 5. Post-hoc analysis revealed that CVI measurements were generally consistent between the two scan patterns across all ETDRS regions, except in the T2 subfield and outer ring. Specifically, the 31-line raster scan measured a significantly smaller CVI than the 12-line radial scan in the T2 subfield (*p* = 0.005) and outer ring (*p* = 0.02).

## Discussion

The most commonly used acquisition patterns for CVI measurements in previous studies are raster and radial scans. This study aimed to determine whether meaningful differences exist between the 12-line radial and 31-line raster scans in measuring CVI, considering their differences in scanning pattern and density. Overall, the results showed that the 12-line radial scan is statistically comparable to the 31-line raster scan for CVI measurements. However, regional discrepancies were observed: the 12-line radial scan tended to yield higher CVI values in the perifoveal region, whereas the 31-line raster scan produced greater CVI values in the central foveal region. This may be explained by differences in the proportion of SA relative to TCA measured by the two scan patterns. In the perifoveal ring, the 12-line radial scan measured a proportionally smaller SA than the 31-line raster scan, resulting in higher CVI values despite smaller absolute values of TCA, LA and SA. Meanwhile, in the central foveal ring, the 31-line raster scan measured a smaller SA relative to TCA, leading to higher CVI values in this region (Supplementary eTable 6).

These regional differences likely arise from the variations in scan density or inter-scan spacing, inherent to each scan pattern. The 31-line raster scan covers a rectangular macular area with evenly spaced B-scans, maintaining a constant inter-scan spacing of 250 μm across the entire scan area due to its parallel arrangement. In contrast, the 12-line radial scan comprises B-scans arranged radially around the foveal centre, each separated by 15°, resulting in a higher scan density at the fovea that decreases gradually with increasing eccentricity. Based on prior methods for estimating radial scan density [32,33], the inter-scan spacing of the 12-line radial scan is approximately 26 μm at a distance of 100 μm from the foveal centre. However, as the distance increases, the spacing becomes wider and eventually exceeds that of the 31-line raster scan beyond 1000 μm from the fovea. As such, the 31-line raster scan is expected to provide more reliable CVI measurements in the perifoveal region due to its greater scan density in the periphery. Conversely, the 12-line radial scan offers nearly ten times the scan density within the central fovea compared with the 31-line raster scan, suggesting that it may yield more accurate CVI measurements in the central foveal region. Similar findings have been reported in previous studies comparing radial and raster scan patterns for the detection of macular pathologies. While both 6-line and 12-line radial scans were found to be comparable to the 25-line raster scan in identifying macular abnormalities, the radial scans demonstrated superior performance in detecting focal vitreomacular traction and full-thickness macular holes, but were less sensitive to extrafoveal or eccentric pathologies than the raster scan [32,33].

Another factor that may contribute to the differing regional performance of the scan patterns is the variation in the number of total data points used for CVI measurements within each ETDRS subfield, as presented in Table 1. These data points were estimated based on the length of the ROI in each B-scan involved, irrespective of the total number of B-scans used. As shown in the table, an equal number of B-scans does not necessarily correspond to an equal number of total pixels (i.e., A-scans) for the two scan patterns. For instance, in the S2 or I2 subfields, CVI measurements from both scan patterns were derived from seven B-scans, but the 12-line radial scan contributed only 896 pixels, compared with 1830 pixels in the 31-line raster scan. Overall, the 31-line raster scan provided more data points for CVI measurements in the perifoveal subfields (S2, I2, T2, N2), whereas the 12-line radial scan offered more data points in the central subfield (1024 pixels vs. 233 pixels for the raster scan). These differences further support the observation that the radial scan may yield more reliable CVI measurements in the central fovea, while the raster scan offers greater precision in the perifovea. In the parafoveal subfields (S1, I1, T1, N1), both scan patterns included a comparable number of total data points, which aligns with the minimal mean differences observed between scan patterns in the Bland-Altman analysis for this region. Therefore, although both scan patterns generally produce comparable CVI measurements, the choice of scan pattern should be made carefully in studies involving regional or topographical analysis of CVI.

Unlike the retinal layers, a universal nomenclature for the choroidal layers has not yet been well established. This lack of standardisation poses challenges when comparing anatomical descriptions of each choroidal layer, particularly the definition of the CSI, across studies involving ChT and CVI measurements. Yiu et al. [29] previously performed ChT measurements using three posterior boundary landmarks: (1) the outer border of the choroidal vessels (vascular ChT), (2) the inner border of the SCL (stromal ChT) and (3) the inner border of the sclera (total ChT), where total ChT differs from stromal ChT only when the SCL is visible. In nearly 60% of their study eyes, the SCL was not visible and a significant mean difference of 17.7 ± 16.0 μm was reported between vascular ChT and stromal or total ChT. However, no previous study has explored the impact of different CSI boundary definitions on CVI measurements. Therefore, the present study compared CVI values obtained using the upper (vascular), middle (vascular–stromal) and lower (stromal) CSI boundaries. The findings revealed mean differences of approximately 1–2% across the ETDRS regions when comparing the upper versus middle and lower versus middle CSI boundaries. Comparisons between the upper and lower CSI boundaries resulted in larger differences of around 2–4%.

The highest CVI values were observed consistently when the upper CSI boundary was used, followed by the middle and lower boundaries. This trend is likely because using the upper CSI boundary includes less hyperreflective SA, resulting in a proportionally larger LA and thus a higher CVI. Conversely, using the lower CSI boundary includes more SA, thereby reducing the CVI. However, such trends were not evident in previous studies. For instance, Sonoda et al. [7], who appear to have used the upper CSI boundary, reported a mean L/C ratio of 65.8%, whereas Agrawal et al. [4], who likely adopted the lower CSI boundary, reported a comparable mean CVI of 65.6% in healthy eyes. The minimal discrepancy between these two studies, despite the likely use of different CSI boundaries, may be attributed to large differences in sample size and participants’ age range, which could have masked the potential impact of CSI delineation on CVI measurements. In addition, a previous study comparing Sonoda’s and Agrawal’s image binarisation techniques reported that CVI values were 2–3% higher than L/C ratios [53]. Apart from potential differences in CSI definitions, which were not clearly specified in previous studies, a key difference between the two techniques is that Sonoda’s method determines the choroidal borders on grayscale OCT images before image binarisation, whereas Agrawal’s method delineates the choroidal borders after image binarisation. Although the latter technique has been more widely adopted in CVI studies, possibly because binarisation is thought to enhance image contrast and provide clearer visualisation of the CSI boundary, the former technique may be more consistent with existing anatomical definitions of the CSI, which are typically based on non-binarised OCT images.

Given that CSI delineation in the present study was based on binarised images, a methodological consideration is that the apparent visibility and location of the CSI may be influenced by the thresholding process, rather than reflecting anatomical boundaries alone. Therefore, additional analyses were conducted to compare CVI measurements obtained from delineating the CSI on binarised versus non-binarised images. As shown in Supplementary eTables 7 and 8, the non-binarised and binarised methods showed the highest agreement and reproducibility when the middle CSI boundary was used, compared with the upper and lower CSI boundaries. Their mean differences were small, being −0.008 mm² for TCA, −0.002 mm² for LA, −0.006 mm² for SA and −0.32% for CVI, although the differences in TCA (*p* = 0.01) and SA (*p* = 0.01) remained statistically significant. Nevertheless, these differences were much smaller than the smallest mean differences observed between the upper and lower CSI boundaries, which were −0.027 mm² for TCA, −0.011 mm² for LA, −0.016 mm² for SA and 2.90% for CVI, as reported earlier in the Results section. These findings further support the use of the middle CSI boundary in the present study. The results also suggest that although the impact of using binarised or non-binarised images for CVI measurements should not be neglected, the use of different CSI boundaries on binarised images may have a greater impact on CVI measurements than whether binarised or non-binarised images were used. Therefore, establishing a standardised and consensus-based definition of the CSI is critical to ensure measurement consistency and enable reliable comparisons across future CVI studies.

On the other hand, topographical variations in CVI were observed across the ETDRS grid in the present study. Specifically, CVI was lowest in the outer nasal subfield, nasal quadrant and outer ring. This regional distribution pattern of CVI was consistent across both the 12-line radial and 31-line raster scan patterns, with no significant differences between the two. However, the current findings contrast with several previous studies that utilised the ETDRS grid to assess the topographical distribution of CVI in young adults, who typically reported the lowest CVI in the central foveal region, with values increasing toward the perifovea and highest in the nasal perifoveal region [17,19,21,54,55]. Like the current study, these investigations also focused on two-dimensional CVI (2D-CVI) measurements. Interestingly, the present results align more closely with studies that examined volumetric or three-dimensional CVI (3D-CVI). For instance, Liu et al. [25] found the highest 3D-CVI at the central fovea and the lowest 3D-CVI in the nasal quadrant among both low-to-moderate and high myopic eyes. Likewise, Luo et al. [56] reported no significant differences in parafoveal 3D-CVI among quadrants in emmetropes, mild myopes and moderate myopes, but observed significantly lower 3D-CVI in the nasal quadrant of high myopes. In the perifoveal region, significant inter-quadrant differences in 3D-CVI were noted across all refractive error groups, with the nasal quadrants consistently showing the lowest 3D-CVI values. The discrepancy in regional patterns observed between 2D-CVI and 3D-CVI across existing studies remains to be elucidated fully. It is worth noting that due to resolution limitations in OCT imaging, 3D-CVI primarily includes medium- and large-sized choroidal vessels [56], while the choriocapillaris, often visualised as a hyporeflective band of vascular LA just beyond Bruch’s membrane in 2D-CVI, is typically excluded due to the difficulty in clearly delineating its boundaries. Nevertheless, as 3D-CVI captures volumetric information about the choroid, it is considered superior to 2D-CVI in reflecting the true choroidal vascular status. Therefore, the topographical patterns observed in 3D-CVI, which are consistent with the current findings, may offer a more representative assessment of the choroidal vasculature.

The present study also revealed that ChT exhibited greater variability than CVI between the 12-line radial and 31-line raster scan patterns across the ETDRS grid, suggesting that ChT may be more influenced by the choice of OCT scan pattern and less reproducible across scan patterns compared with CVI. Similar observations have been reported in previous large-scale, population-based studies. Agrawal et al. [4] found a lower CoV for CVI than for subfoveal ChT (3.55 vs. 40.30%) and highlighted that CVI was less affected by physiological factors such as AL, IOP, LA and SBP, all of which were closely associated with subfoveal ChT. Wang et al. [23] also reported a smaller CoV for CVI compared with ChT (5.33 vs. 24.29%) in a cohort of 380 Chinese individuals aged 8–30 years. These findings support the notion that CVI is a relatively more stable and robust biomarker and should be used alongside ChT when assessing choroidal changes in future studies.

To our knowledge, this is the first study to examine the influence of SD-OCT scan pattern and CSI delineation on CVI measurements. Unlike prior studies that often relied on a single horizontal and/or vertical OCT B-scan, this investigation assessed CVI across the ETDRS grid, providing a more comprehensive topographical evaluation. However, several limitations should be acknowledged. First, this study included only healthy young adults, which may limit the generalisability of the findings to other age groups and individuals with chorioretinal pathology. Younger children and older adults may show lower compliance with raster scans due to longer acquisition times, potentially leading to poorer agreement in CVI measurements between radial and raster scans. In addition, poorer visualisation of the CSI has been reported in younger individuals [57], which may influence the effect of different CSI definitions on CVI values across age groups. Future studies, including a broader age range and pathological populations, are therefore warranted. Second, only a single SD-OCT device was used. While previous studies have reported good agreement between CVI values derived from SD-OCT and SS-OCT [28], validation across multiple OCT devices would strengthen further the generalisability of the findings. Third, CVI measurements were manually performed using ImageJ software by two experienced examiners, which may introduce potential intra- and inter-examiner variability. Fourth, although the current sample size was sufficient to achieve at least 80% statistical power for the primary comparison between scan patterns, it may be inadequate for subgroup analyses involving multiple comparisons across the ETDRS regions, thereby increasing the risk of a Type II error. Additionally, the relatively small cohort and wide range of refractive errors may introduce potential confounding effects. As CSI delineation can be more challenging in thicker choroids (e.g., emmetropic or low myopic eyes) and easier in thinner choroids in high myopic eyes, the potential influence of refractive error on the findings cannot be excluded. Future studies with larger sample sizes, including individuals with emmetropia or lower refractive errors, are needed to validate these findings further. Fifth, this study did not account for the presence or absence of SCL, which may influence the CSI definition and further contribute to variability in CVI measurements. However, previous studies have reported that the SCL is visible in only approximately 40% of the 74 examined eyes [29] and in about 5% of healthy emmetropic and myopic eyes [58], suggesting that the SCL is not a universally present or reliable landmark for CSI delineation. Furthermore, although the middle CSI boundary tested in this study may lack a clear histological basis or widespread acceptance in the literature, its intergrader reliability (ICC: 0.943–0.987) was comparable to that reported in a previous study [59], which showed higher intergrader reliability for stromal ChT (ICC: 0.959–0.980) than for vascular and total ChT. Lastly, due to the lack of consensus in the literature regarding the exact anatomical location of the CSI, we were unable to determine which of the three tested CSI definitions in this study offers more accurate CVI values. Nonetheless, the present results underscore the need for standardisation in CSI delineation, as variability in its definition leads to meaningful discrepancies in CVI measurements.

## Conclusions

The 12-line radial scan provides CVI measurements with comparable precision to the 31-line horizontal raster scan, while requiring less chair time, making it a more practical option for routine clinical examination, particularly in paediatric populations. However, careful selection of scan pattern remains essential for studies involving regional or topographical CVI assessment, as the radial scan offers greater precision in the central fovea, whereas the raster scan is more reliable in the perifoveal region and beyond. Additionally, the lack of a standardised definition for the CSI presents a challenge for consistent CVI measurements. Establishing a consensus on CSI delineation across examiners and studies is imperative to ensure reproducibility and to facilitate meaningful comparisons in future CVI research.

## Supplementary Information

Supplementary Information

## Data Availability

The data that support the findings of this study are available from the corresponding author upon reasonable request.
