# Supplementary material for: Impact of Optical Coherence Tomography Scan Pattern and Chorio-Scleral Interface Delineation on Choroidal Vascularity Index Measurements
Source: Ophthalmic Physiol Opt. 2026 Aug 15;46(5):1218–28. doi: 10.1007/s44402-026-00159-4 (PMC13545132; doi:10.1007/s44402-026-00159-4)
Supplement: Supplementary file 1 [file 44402_2026_159_MOESM1_ESM.pdf]

Supplementary Information

Journal Name

Ophthalmic and Physiological Optics

Manuscript Title

Impact of optical coherence tomography scan pattern and chorio-scleral interface delineation on choroidal vascularity index measurements

Authors' Names

Kryshell Yu Qi Wong[1], Rachel Ka Man Chun[1,2,3], David Alonso-Caneiro[4,5], Yan Pui Chan[2] and Andrew Kwok Cheung Lam[1,2,3]

Affiliations

[1]Centre for Myopia Research, School of Optometry, The Hong Kong Polytechnic University, Hung Hom, Kowloon, Hong Kong.
[2]Centre for Eye and Vision Research (CEVR), 17W Hong Kong Science Park, Sha Tin, Hong Kong.
[3]Research Centre for SHARP Vision (RCSV), The Hong Kong Polytechnic University, Hung Hom, Kowloon, Hong Kong.
[4]School of Science, Technology and Engineering, University of Sunshine Coast, Petrie, Queensland, Australia.
[5]Queensland University of Technology (QUT), Contact Lens and Visual Optics Laboratory, Centre for Vision and Eye Research, Optometry and Vision Science, Kelvin Grove, Queensland, Australia.

Corresponding Author

Andrew Kwok Cheung Lam, Centre for Myopia Research, School of Optometry, The Hong Kong Polytechnic University, Hung Hom, Kowloon, Hong Kong.
Email: andrew.kc.lam@polyu.edu.hk

eTable 1. Mean differences in TCA derived from three different CSI boundaries.

ETDRS	Mean Difference [95% LoA]		
	**Upper vs. Middle CSI**	**Middle vs. Lower CSI**	**Upper vs. Lower CSI**
C	–0.0169 [–0.0404 to 0.0067]	–0.0196 [–0.0473 to 0.0082]	–0.0365 [–0.0722 to –0.0007]
S1	–0.0152 [–0.0334 to 0.0030]	–0.0138 [–0.0388 to 0.0112]	–0.0290 [–0.0603 to 0.0022]
I1	–0.0125 [–0.0288 to 0.0038]	–0.0148 [–0.0393 to 0.0096]	–0.0273 [–0.0559 to 0.0012]
T1	–0.0160 [–0.0335 to 0.0015]	–0.0193 [–0.0450 to 0.0063]	–0.0353 [–0.0697 to –0.0010]
N1	–0.0151 [–0.0341 to 0.0039]	–0.0170 [–0.0438 to 0.0098]	–0.0321 [–0.0689 to 0.0048]
S2	–0.0224 [–0.0462 to 0.0013]	–0.0227 [–0.0634 to 0.0180]	–0.0451 [–0.0914 to 0.0011]
I2	–0.0192 [–0.0438 to 0.0054]	–0.0264 [–0.0613 to 0.0085]	–0.0456 [–0.0930 to 0.0017]
T2	–0.0231 [–0.0507 to 0.0046]	–0.0273 [–0.0660 to 0.0114]	–0.0504 [–0.1011 to 0.0003]
N2	–0.0243 [–0.0538 to 0.0052]	–0.0301 [–0.0681 to 0.0080]	–0.0543 [–0.1084 to –0.0003]

Note. All pairwise differences were statistically significant, paired samples *t*-tests with Bonferroni correction, $p<.01$.

Abbreviations: CSI, chorio-scleral interface; LoA, limits of agreement

eTable 2. Mean differences in LA derived from three different CSI boundaries.

ETDRS	Mean Difference [95% LoA]		
	**Upper vs. Middle CSI**	**Middle vs. Lower CSI**	**Upper vs. Lower CSI**
C	–0.0061 [–0.0208 to 0.0086]	–0.0097 [–0.0250 to 0.0056]	–0.0158 [–0.0364 to 0.0048]
S1	–0.0063 [–0.0178 to 0.0053]	–0.0064 [–0.0191 to 0.0063]	–0.0127 [–0.0295 to 0.0041]
I1	–0.0045 [–0.0132 to 0.0042]	–0.0060 [–0.0193 to 0.0072]	–0.0105 [–0.0264 to 0.0053]
T1	–0.0062 [–0.0170 to 0.0046]	–0.0080 [–0.0217 to 0.0057]	–0.0142 [–0.0330 to 0.0046]
N1	–0.0053 [–0.0159 to 0.0052]	–0.0067 [–0.0211 to 0.0078]	–0.0120 [–0.0337 to 0.0097]
S2	–0.0079 [–0.0243 to 0.0086]	–0.0101 [–0.0297 to 0.0094]	–0.0180 [–0.0457 to 0.0098]
I2	–0.0077 [–0.0207 to 0.0053]	–0.0111 [–0.0310 to 0.0089]	–0.0187 [–0.0460 to 0.0085]
T2	–0.0078 [–0.0238 to 0.0082]	–0.0110 [–0.0308 to 0.0088]	–0.0188 [–0.0471 to 0.0095]
N2	–0.0098 [–0.0245 to 0.0049]	–0.0124 [–0.0295 to 0.0046]	–0.0222 [–0.0489 to 0.0045]

Note. All pairwise differences were statistically significant, paired samples *t*-tests with Bonferroni correction, $p<.01$.

Abbreviations: CSI, chorio-scleral interface; LoA, limits of agreement

eTable 3. Mean differences in SA derived from three different CSI boundaries.

ETDRS	Mean Difference [95% LoA]		
	**Upper vs. Middle CSI**	**Middle vs. Lower CSI**	**Upper vs. Lower CSI**
C	–0.0108 [–0.0224 to 0.0008]	–0.0100 [–0.0263 to 0.0065]	–0.0207 [–0.0420 to 0.0006]
S1	–0.0090 [–0.0181 to 0.0001]	–0.0074 [–0.0229 to 0.0081]	–0.0163 [–0.0362 to 0.0036]
I1	–0.0080 [–0.0183 to 0.0023]	–0.0088 [–0.0228 to 0.0051]	–0.0168 [–0.0348 to 0.0011]
T1	–0.0098 [–0.0189 to –0.0007]	–0.0113 [–0.0252 to 0.0026]	–0.0211 [–0.0402 to –0.0020]
N1	–0.0097 [–0.0207 to 0.0012]	–0.0103 [–0.0243 to 0.0037]	–0.0201 [–0.0390 to –0.0012]
S2	–0.0146 [–0.0283 to –0.0009]	–0.0126 [–0.0357 to 0.0105]	–0.0272 [–0.0495 to –0.0048]
I2	–0.0115 [–0.0251 to 0.0021]	–0.0154 [–0.0330 to 0.0023]	–0.0269 [–0.0509 to –0.0029]
T2	–0.0153 [–0.0307 to 0.0001]	–0.0163 [–0.0374 to 0.0048]	–0.0316 [–0.0576 to –0.0056]
N2	–0.0145 [–0.0329 to 0.0039]	–0.0176 [–0.0418 to 0.0066]	–0.0321 [–0.0652 to 0.0009]

Note. All pairwise differences were statistically significant, paired samples *t*-tests with Bonferroni correction, $p<.01$.

Abbreviations: CSI, chorio-scleral interface; LoA, limits of agreement

eTable 4. Mean ChT values and the degree of variation and agreement between the 12-line radial and 31-line raster scan patterns across ETDRS subfields, quadrants, and rings.

**ETDRS**	**Choroidal Thickness, µm**			
	**Mean ± SD**			**Bland-Altman**
	**12-line radial**	**31-line raster**	**CoV, %**	**Mean Difference [95% LoA]**
Subfields				
C	300.67 ± 100.90	304.77 ± 106.79	2.84	4.10 [–29.28 to 37.48]**
S1	295.58 ± 84.82	303.19 ± 103.01	5.63	7.62 [–55.02 to 70.25]**
I1	293.30 ± 87.64	303.72 ± 108.72	5.48	10.42 [–57.86 to 78.71]**
T1	305.33 ± 86.30	314.12 ± 101.93	4.91	8.79 [–41.01 to 58.60]**
N1	231.21 ± 90.13	285.14 ± 107.08	14.82	53.93 [–9.09 to 116.95]**
S2	296.83 ± 67.84	304.75 ± 96.06	7.63	7.92 [–68.72 to 84.56]**
I2	266.47 ± 70.71	297.90 ± 92.83	8.15	31.43 [–36.80 to 99.67]**
T2	291.78 ± 70.54	311.46 ± 93.07	6.24	19.68 [–40.77 to 80.13]**
N2	180.06 ± 62.23	241.97 ± 97.54	19.10	61.91 [–27.25 to 151.07]**
Quadrants				
Superior	296.20 ± 75.50	303.97 ± 98.67	6.10	7.77 [–57.10 to 72.64]**
Inferior	279.88 ± 78.20	300.81 ± 100.04	5.91	20.93 [–40.80 to 82.66]**
Temporal	298.56 ± 78.01	312.79 ± 97.18	5.02	14.24 [–37.71 to 66.18]**
Nasal	205.64 ± 75.31	263.56 ± 101.70	16.64	57.92 [–12.25 to 128.09]**
Rings				
Central foveal	300.67 ± 100.90	304.77 ± 106.79	2.84	4.10 [–29.28 to 37.48]**
Inner	281.35 ± 83.44	301.55 ± 103.57	5.16	20.19 [–31.02 to 71.40]**
Outer	258.79 ± 62.65	289.02 ± 91.64	8.05	30.24 [–36.23 to 96.70]**

Statistical significance was determined using one sample *t*-test, *p<.05, **p<.01.
Abbreviations: CoV, coefficient of variation; LoA, limits of agreement; SD, standard deviation.

eTable 5. Estimated marginal means and 95% confidence intervals of CVI across ETDRS subfields, quadrants and rings.

**ETDRS**	**Mean (SE)**[‡]	**95% Confidence Interval**	
		**Lower Bound**	**Upper Bound**
Subfields			
C	65.72 (0.38) [e, f, i]	64.94	66.50
S1	65.61 (0.29) [c, d, e, f, i]	65.01	66.21
I1	66.41 (0.46) [b, e, f, g, h, i]	65.47	67.35
T1	66.30 (0.42) [b, f, g, i]	65.44	67.15
N1	66.50 (0.46) [a, b, f, g, h, i]	65.55	67.45
S2	64.71 (0.31) [a, b, c, d, e, g, h]	64.08	65.35
I2	65.30 (0.29) [c, d, e, f, i]	64.71	65.89
T2	65.66 (0.32) [c, e, f, i]	65.00	66.31
N2	64.41 (0.53) [a, b, c, d, e, g, h]	63.32	65.49
Quadrants			
Superior	65.45 (0.44)	64.54	66.37
Inferior	65.86 (0.32)	65.19	66.53
Temporal	65.98 (0.33)	65.31	66.65
Nasal	65.16 (0.27) [†‡]	64.61	65.71
Rings			
Central foveal	65.72 (0.38)	64.94	66.50
Inner	66.21 (0.35)	65.49	66.93
Outer	65.02 (0.29) [§ϕ]	64.42	65.61

[‡]Estimated marginal means (standard error) derived from two-way repeated measures ANOVA. Post-hoc comparisons with Bonferroni correction indicated statistically significant differences ($p<.05$) in CVI compared to: [a]C, [b]S1, [c]I1, [d]T1, [e]N1, [f]S2, [g]I2, [h]T2, [i]N2 subfields; [†]Inferior quadrant, [‡]temporal quadrant; [§]Central circle, [ϕ]inner ring.

eTable 6. Mean TCA, LA, and SA values and the degree of variation and agreement between the 12-line radial and 31-line raster scan patterns across the central-foveal, parafoveal and perifoveal rings.

ETDRS ring	Mean ± SD		CoV, %	Bland-Altman
	12-line radial	31-line raster		Mean Difference [95% LoA]
**Total choroidal area (TCA), mm^2**				
Central foveal	0.323 ± 0.115	0.290 ± 0.105	7.78	–0.033 [–0.078 to 0.013]**
Inner (parafoveal)	0.316 ± 0.108	0.354 ± 0.123	7.74	0.037 [–0.002 to 0.077]**
Outer (perifoveal)	0.447 ± 0.140	0.652 ± 0.209	26.23	0.205 [0.060 to 0.351]**
**Luminal area (LA), mm^2**				
Central foveal	0.211 ± 0.076	0.191 ± 0.068	7.35	–0.021 [–0.054 to 0.012]**
Inner (parafoveal)	0.209 ± 0.070	0.233 ± 0.079	7.74	0.024 [–0.003 to 0.051]**
Outer (perifoveal)	0.293 ± 0.092	0.422 ± 0.135	25.36	0.129 [0.037 to 0.221]**
**Stromal area (SA), mm^2**				
Central foveal	0.111 ± 0.040	0.099 ± 0.037	9.00	–0.012 [–0.031 to 0.007]**
Inner (parafoveal)	0.108 ± 0.039	0.121 ± 0.044	7.84	0.013 [–0.005 to 0.031]**
Outer (perifoveal)	0.154 ± 0.049	0.230 ± 0.074	27.90	0.076 [0.021 to 0.132]**

Statistical significance was determined using one sample *t*-test, **p*<.05, ***p*<.01.
Abbreviations: CoV, coefficient of variation; LoA, limits of agreement; SD, standard deviation.

eTable 7. Agreement and reproducibility between binarized and non-binarized methods performed by Examiner 1 (KW), using three CSI boundary definitions for the binarized method.

Outcome	ICC [95% CI]	CoV, %	Mean Difference [95% LoA]
Non-binarized vs. Binarized (upper CSI boundary)			
TCA, mm^2	0.981 [0.675–0.995]	3.49	0.017 [–0.009 to 0.043]**
LA, mm^2	0.987 [0.877–0.996]	2.70	0.008 [–0.007 to 0.024]**
SA, mm^2	0.965 [0.480–0.991]	8.67	0.009 [–0.004 to 0.021]**
CVI, %	0.339 [0.018–0.633]	3.10	–2.097 [–9.099 to 4.906]**
Non-binarized vs. Binarized (middle CSI boundary)			
TCA, mm^2	0.989 [0.968–0.995]	3.30	–0.008 [–0.037 to 0.021]*
LA, mm^2	0.990 [0.960–0.997]	3.10	–0.002 [–0.019 to 0.016]
SA, mm^2	0.973 [0.941–0.988]	5.62	–0.006 [–0.023 to 0.010]*
CVI, %	0.866 [0.723–0.939]	1.54	–0.319 [–3.531 to 2.893]
Non-binarized vs. Binarized (lower CSI boundary)			
TCA, mm^2	0.941 [0.359–0.985]	6.90	–0.031 [–0.079 to 0.016]**
LA, mm^2	0.963 [0.612–0.990]	5.18	–0.016 [–0.043 to 0.011]**
SA, mm^2	0.885 [0.155–0.969]	10.19	–0.016 [–0.039 to 0.007]**
CVI, %	0.779 [0.156–0.926]	2.04	1.634 [–1.459 to 4.728]**

Note. For the non-binarized method, the CSI was delineated on non-binarized grayscale OCT images without further adjustment after image binarization. For the binarized method, the CSI boundary was further adjusted after image binarization. To assess agreement and reproducibility between the two methods, Examiner 1 performed CVI measurements on 25 OCT images randomly selected from the 12-line radial scans. Mean differences and 95% LoA were determined using Bland–Altman analysis. Statistical significance was determined using paired samples t-test, *p<.05, **p<.01.
Abbreviations: CI, confidence interval; CoV, coefficient of variation; CSI, chorio-scleral interface; CVI, choroidal vascularity index; ICC, intraclass correlation coefficient; LA, luminal area; LoA, limits of agreement; SA, stromal area; TCA, total choroidal area.

eTable 8. Agreement and reproducibility between binarized and non-binarized methods performed by Examiner 2, a masked examiner (PC), using the middle CSI boundary only for the binarized method.

Outcome	ICC [95% CI]	CoV, %	Mean Difference [95% LoA]
TCA, mm^2	0.989 [0.956–0.996]	3.96	–0.010 [–0.036 to 0.017]*
LA, mm^2	0.989 [0.976–0.995]	2.93	–0.002 [–0.023 to 0.019]
SA, mm^2	0.953 [0.782–0.984]	6.10	–0.008 [–0.027 to 0.011]*
CVI, %	0.918 [0.507–0.975]	1.21	–0.981 [–2.907 to 0.946]

Note. For the non-binarized method, the CSI was delineated on non-binarized grayscale OCT images without further adjustment after image binarization. For the binarized method, the CSI boundary was further adjusted after image binarization. To assess agreement and reproducibility between the two methods, Examiner 2 performed CVI measurements on the same set of 25 OCT images. Mean differences and 95% LoA were determined using Bland–Altman analysis. Statistical significance was determined using paired samples t-test, *p<.05, **p<.01.
Abbreviations: CI, confidence interval; CoV, coefficient of variation; CSI, chorio-scleral interface; CVI, choroidal vascularity index; ICC, intraclass correlation coefficient; LA, luminal area; LoA, limits of agreement; SA, stromal area; TCA, total choroidal area.
